# Construction and optimization of vending machine decision support system based on improved C4.5 decision tree

**DOI:** 10.1016/j.heliyon.2024.e25024

**Published:** 2024-01-23

**Authors:** Ping Li, Fang Xiong, Xibei Huang, Xiaojun Wen

**Affiliations:** School of Information and Mechatronic Engineering, Hunan International Economics University, Changsha, 410205, China

**Keywords:** Machine learning, Decision support system, Decision tree, Neural network, Vending system

## Abstract

The intensification of market competition makes refined operation management become the focus of attention of major manufacturers. As an important branch of artificial intelligence (AI), machine learning (ML) plays a key role in it, and has its application prospect in various systems. Based on this situation, this paper takes vending machines as the research object. On the one hand, the product classification model of vending machine is constructed based on decision tree algorithm. On the other hand, based on neural network (NN), the sales forecast model of vending machines is built. Finally, based on the above research, the theoretical framework of decision support system (DSS) for vending machines is constructed. The research shows that: (1) The accuracy of C4.5 algorithm can reach 87 % at the highest and 68 % at the lowest. The accuracy of the improved C4.5 algorithm can reach 87 % at the highest and 67 % at the lowest, with little difference between them. (2) The maximum running time of the improved C4.5 algorithm is about 5500 ms, and the minimum is close to 1 ms. In addition, the running time of all seven datasets is better than that of the unmodified algorithm. (3) When the back propagation neural network (BPNN) is used to forecast the sales of vending machines, the curve of the predicted data basically coincides with the curve of the actual data, which shows that its accuracy is high. This paper aims to build a convenient and secure DSS by taking vending machines as an example. In addition, this paper also uses reinforcement learning to optimize the research methods of this paper. It can further optimize the performance and efficiency of vending machines and provide better service experience for customers. Meanwhile, the use of reinforcement learning can make the whole system more intelligent and adaptive to better cope with the changing market environment.

## 1 Introduction

The Decision Support System (DSS) is a human-computer interaction (HCI) system that provides intelligent decision support for decision-makers for semi-structured decision problems. It uses computer, information, and simulation technology and integrates management science, operations research, cybernetics, and behavioral science to provide accurate guarantees for various decision support [1]. In short, a DSS is a system that provides auxiliary decision support for users through HCI based on data and models. DSSs emphasize the support of management decisions rather than the automation of decisions [2]. A lightweight Internet of Things (IoT) DSS is designed to realize the efficient and intelligent operation and management of vending machines. It is meaningful to formulate optimal sales plans for managers and provide scientific and data-based auxiliary support in decision management through some intelligent algorithms.

Machine Learning (ML), the most important part of the field of Artificial Intelligence (AI), is developing rapidly and has gradually penetrated various industries [3]. ML realizes the learning behavior of the human brain through simulation so that the machine “masters” new knowledge, continuously improves its existing knowledge structure, and improves its learning efficiency. The main research areas include supervised learning, unsupervised learning, and reinforcement learning. Vending machines have developed for decades since their birth in the 1960s and 1970s. Its manufacturing process and precision have been greatly improved, and its functionality has gradually diversified [4]. From the perspective of development history, China began to introduce vending machines in the 1990s and developed them independently. The functions had also changed significantly with the addition of many new technologies such as the IoT, mobile payment, and intelligent management [5].

Based on this, the paper firstly introduces the basic knowledge of ML algorithms, including some common supervised learning and data mining algorithms. Secondly, a commodity classification model based on decision tree algorithm is constructed. In addition, a sales forecasting model based on NN is constructed to predict the sales volume of vending machines in the next year. Finally, the vending machine DSS is constructed, and the system model is comprehensively optimized by reinforcement learning to analyze its test results. The innovation of this paper lies in the application of NN technology to ML and decision tree algorithm to data mining, and the sales forecast model and commodity classification model of vending machines are constructed, respectively. It combines AI and ML technology, and provides a theoretical reference for DSSs with high stability and security. This is a fusion innovation of ML technology and DSS. It is also a useful attempt to improve the management level and technological innovation of sales and traditional manufacturing enterprises.

The main contribution of this paper is to deeply discuss the performance of product classification model based on different algorithms, sales forecasting model based on neural network, and reinforcement learning optimization model. By applying C4.5 decision tree and its improved algorithm, this paper studies and compares the accuracy and running time of different datasets, and shows the obvious advantages of the improved algorithm in running time. In addition, the sales forecasting model based on BPNN shows the ability to predict the actual sales trend with high accuracy. Finally, reinforcement learning technology is introduced to optimize the system, and the results show that the optimized system has significantly improved accuracy and response time, which provides strong support for the overall improvement of system performance. Generally speaking, this paper provides in-depth empirical analysis and strong data support for related fields, which has important theoretical and practical value.

## 2 Theoretical basis and experimental design

### 2.1 Literature review

As the most important part of AI, ML is developing rapidly and gradually permeating into various industries. Researchers have put forward many practical application cases based on ML algorithm. For example, Amazon has established a complete personalized recommendation system based on Apriori algorithm and a large number of user data. According to the data, at least 20 % of Amazon's sales come from the recommendation system. The semantic real-time translation system developed by Iflytek through deep learning has reached CET-6, and can even translate dialects such as Henan dialect and Cantonese. Google's Alpha Go beat Li Shishi and Ke Jie, the world's top Go masters, pushed AI to a new height.

As the most important part of AI, ML is developing rapidly and has gradually penetrated various industries [3,6]. Amazon has established a complete personalized recommendation system based on the Apriori algorithm combined with the company's massive user data. According to data, at least 20 % of Amazon's sales come from recommender systems [7]. In speech translation, the semantic real-time translation system made by iFLYTEK Co., Ltd. using deep learning (DL) has achieved the level of English level six and can even translate dialects, such as Henan and Cantonese [8]. The most sensational event in ML is that Google's Alpha Go defeats the world's top Go masters, Lee Sedol and Ke Jie, bringing AI to a new level [9]. Some researchers have discussed some real-life applications of ML algorithms in depth. Kamran and Shahani (2022) [10] proposed a new idea to build an underground coal mine fire prediction DSS using several ML techniques to predict the mine fire level. They proposed a data mining-based DSS using decision trees and artificial neural networks as a hybrid approach to estimate marketing strategies. Rani et al. (2021) [11] proposed a hybrid DSS based on ML algorithms. The system could aid in the early detection of heart disease based on the patient's clinical parameters. Arvind et al. (2021) [12] developed an ML algorithm for predicting the diagnosis of patients. Naranjo et al. (2021) [13] studied many ML algorithms in medical imaging diagnosis to assist doctors in scientific decision-making. The research and rapid development of ML algorithms provide the basis for constructing intelligent DSSs. However, since machines are only used to identify specific choices, specifying options or variations based on human behavior is large. In addition, machines mainly select the right decisions. Still, there may be situations where machines cannot make optimal decisions, and a fully automated IoT DSS requires much research and analysis.

To sum up, the research and rapid development of ML algorithms provide a foundation for building an intelligent DSS. However, because machines can only recognize specific choices, it is difficult to specify options or variants based on human behavior. In addition, machines mainly choose the right decision, but in some cases, they may not be able to make the best decision. Fully automated DSS of the IoT needs more research and analysis. Therefore, when building intelligent DSS, further research and exploration are still needed to overcome these limitations.

### 2.2 Machine learning algorithm and analysis of HCI behavior

#### 2.2.1 Machine learning

**Fig. 1:**
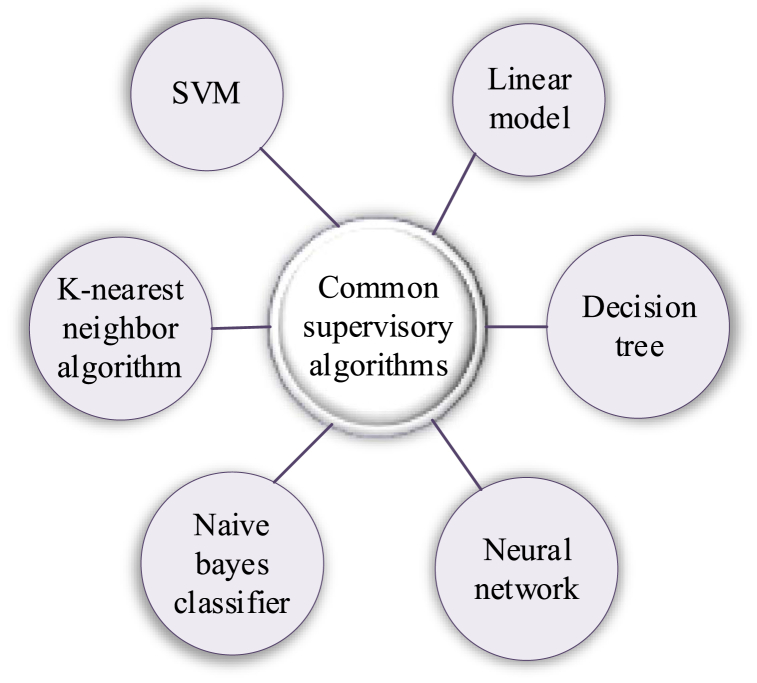
Common supervised algorithms.

There are different ways to model a problem depending on the data type. In ML, there are several main ways of learning. Categorizing algorithms according to their learning styles allows people to consider when modeling and algorithm selection can choose the most appropriate algorithm based on the input data to obtain the best results. Six common supervised algorithms are shown in Fig. 1.

Fig. 1 shows several common machine supervision algorithms. Among them, support vector machine (SVM) is suitable for high-dimensional space and complex decision boundaries, but it is sensitive to large-scale datasets and noise. Linear model is simple and efficient, but it is weak in expressing nonlinear relations. K nearest neighbor algorithm performs well in pattern recognition, but it is sensitive to noise and high-dimensional data [14]. Decision trees are easy to understand and explain, but there are challenges in dealing with continuous data and over-fitting. Naive Bayesian classifier is suitable for text classification and other scenarios, but it is sensitive to the correlation among features [15]. Neural network is powerful in dealing with complex patterns and large-scale data, but it needs a lot of data and computing resources and lacks interpretability.

Based on the above contents, this paper comprehensively considers the diversity of ML methods, and finally chooses neural network and C4.5 decision tree for follow-up research to make full use of their respective advantages. Neural network shows its powerful performance in dealing with complex patterns such as sales forecast, and can capture nonlinear relationships and patterns in large-scale data, providing high prediction accuracy. On the other hand, in the product classification model, C4.5 decision tree is adopted, which has good interpretability and processing ability of discrete data, which is helpful to understand and explain the decision-making process of product classification. This strategy of comprehensively using different ML methods aims to improve the system performance and interpretability, and provide comprehensive support for the optimization and promotion of HCI system.

In unsupervised learning, the data is not identified [16]. The purpose of learning is to determine the internal characteristics of the data. In practical applications, labeling is time-consuming and labor-intensive. Unsupervised learning has a high degree of automation, so it has advantages [17]. Commonly used unsupervised algorithms in engineering practice include clustering algorithms, association rule algorithms, NNs, and DL [18].

In the era of big data, data warehouses, databases, Internet information, or public datasets hide information needed for industrial production and business decision-making. Mining interesting, useful, implicit, previously unknown, and potentially useful patterns or knowledge from this information is data mining [19]. Classification and prediction are two forms of data mining. Classification is about extracting and classifying subsets of datasets that have commonalities, while prediction is about knowing future trends.

**Fig. 2:**
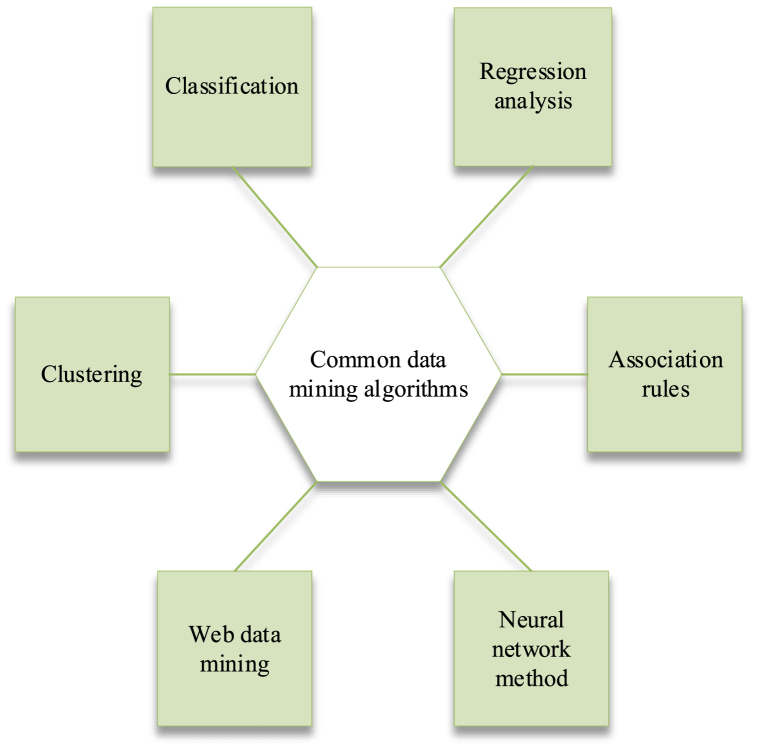
Commonly used data mining algorithms.

Data mining is used for data analysis, and the commonly used methods are shown in Fig. 2.

For the methods used in the sales forecast here, the following mainly introduces the preprocessing technology, the classification method, and the prediction method.

##### 2.2.1.1 Preprocessing

In reality, raw data often have various problems. The training of the model cannot begin without preprocessing the raw data. Common numerical data preprocessing methods consist of missing value processing, continuous feature discretization, discrete feature encoding, normalization, and normalization [20].

##### 2.2.1.2 Prediction method

Assuming that historical data and future data obey similar statistical laws, the laws found in historical data also apply to the future. The inherent laws can be found to predict the future through the construction and modeling of historical data [21].

##### 2.2.1.3 Classification method

Classification is to find out the common characteristics of a group of data objects in the database and divide them into different classes according to the classification pattern. Its purpose is to map data items in the database to a given category through a classification model [22].

#### 2.2.2 Machine learning and HCI

HCI refers to the interaction between human users and computer systems in the process of information exchange and operation between people and computer systems. This includes how users interact with the computer interface, use input devices (such as keyboard, mouse, touch screen, etc.) to operate, and how the system responds to user input. In the field of HCI, ML is widely used in improving user experience, personalized recommendation, natural language processing and so on, which enables computer systems to better understand and adapt to users' needs and behaviors. On the one hand, ML technology enables computer systems to acquire knowledge, patterns and laws from a large number of data, thus realizing more intelligent behavior and decision-making. On the other hand, the study of HCI also provides guidance for ML, and provides useful information for model design and optimization through in-depth understanding of user behavior, psychology, and preferences. This interaction promotes innovation, facilitates the development of technology, and plays an increasingly important role in daily life, work, and entertainment.

### 2.3 Product classification model based on decision tree algorithm

#### 2.3.1 C4.5 decision tree

The C4.5 algorithm is an extension and optimization of the ID3 algorithm. The main improvement is to use the information gain rate to select decision attributes. The C4.5 algorithm solves the problem that the ID3 algorithm tends to select attributes with multiple attribute values as split attributes through information gain. The computational complexity is reduced, and the efficiency of building decision trees is improved. In addition, there are many decision tree algorithms, such as the Rain Forest algorithm [23] and the fuzzy decision tree method [24]. These algorithms all improve C4.5 to a certain extent, and each has its advantages and limitations. In general, the basic ideas have not changed, and no innovative changes have occurred. The top-down recursive method has been adopted, and the basic steps of the algorithm have not fundamentally changed. Since the C5.0 algorithm is mainly provided to commercial users, the C4.5 algorithm is studied here.

The selection of the splitting attribute is the key to the decision tree production process, which determines the performance and structure of the generated decision tree. The criterion for splitting attribute selection is the fundamental difference between decision tree algorithms. The ID3 algorithm uses information gain as the basis for judging the split attribute, while the C4.5 algorithm uses the information gain rate as the attribute for dividing subsets [25].

Information gain is defined as the difference in entropy before and after the change, while entropy is defined as the expected value of information. The information is defined as follows: if the frequency of the classification label *x*_*i*_ in the sample set *S* is recorded as *p*(*x*_*i*_), the information of *x*_*i*_ is defined as -*log*_2_(*x*_*i*_) [26].

The entropy of the sample set before splitting is:

(1) $$E\left(S\right)=-\sum_{i=1}^{N}px_{i}\;\log_{2}\;p\left(x_{i}\right)$$

In Eq. (1), *N* is the number of classification labels.

The “split information” of attribute *A* is expressed as:

(2) $${SplitInfo}_{A}\left(S\right)=-\sum_{j=1}^{m}\frac{{|S}_{j}|}{\left|S\right|}\log_{2}\frac{\left|S_{j}\right|}{\left|S\right|}$$

In Eq. (2), the training dataset *S* is divided into m sub-datasets by the attribute value of attribute *A*. $S_{j}$ represents the number of samples in the *j*th sub-dataset. *S* represents the total number of samples in the dataset before division.

The entropy of the sample set after splitting by attribute *A* is in Eq. (3):

(3) $$E_{A}\left(S\right)=-\sum_{j=1}^{m}\frac{\left|S_{j}\right|}{\left|S\right|}E\left(S_{j}\right)$$

The information gain of the sample set after splitting by attribute *A* is in Eq. (4):

(4) $$InfoGain\left(S,A\right)=E\left(S\right)-E_{A}\left(S\right)$$

The information gain rate of the sample set after splitting by attribute *A* is in Eq. (5):

(5) $$InfoGainRation\left(S,A\right)=\frac{InfoRation\left(S,A\right)}{{SplitInfo}_{A}\left(S\right)}$$

When the C4.5 algorithms are used to construct a decision tree to classify data, the tree bifurcation is the next step according to the attribute with the largest information gain rate. Subsequently, in the recursive phase, the information gain rate of the attribute will become small as the tree continues to fork. In the later stage, the attribute with a large information gain rate is still selected as the splitting attribute.

**Fig. 3:**
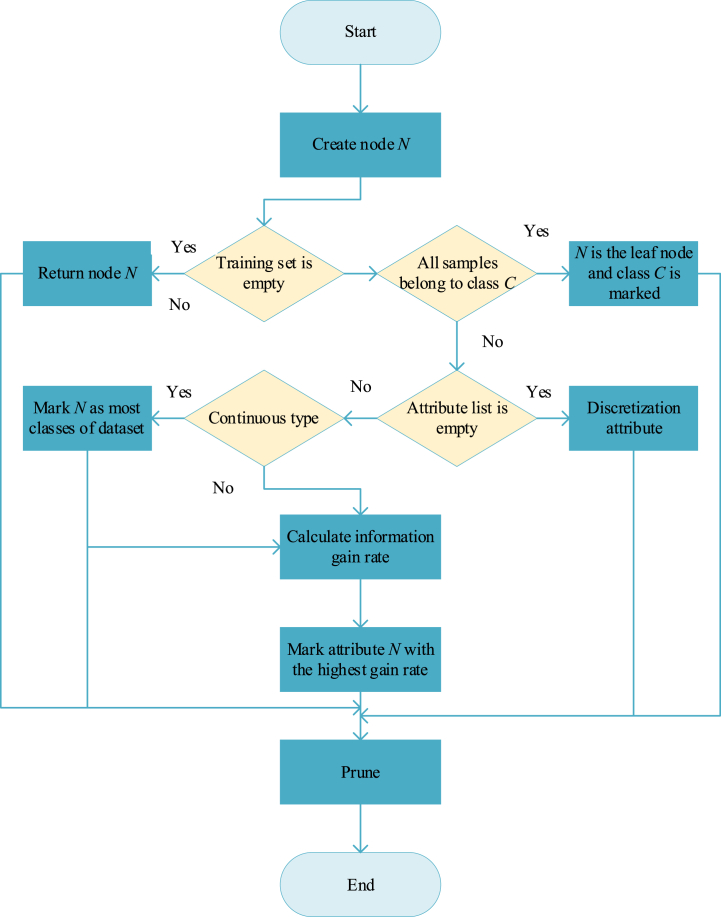
C4.5 algorithm flow.

The operation steps of the C4.5 decision tree algorithm are demonstrated in Fig. 3.

1) At the beginning, all data are in the same root node.
2) First, it is necessary to determine whether there is a split attribute at this time. If it is unique or empty, it means that the entire decision tree has been constructed, and the classification result has been obtained. If there are multiple split attributes, the information gain rate of each attribute should be calculated. In addition, the largest one is selected as the split condition, and the sample set is further divided.
3) Then, the split subset is regarded as a new tree to continue to calculate the information gain rate of each attribute and divide. If the subsample set is empty or cannot be split any further, the classification is complete. On the contrary, if the recursion continues, the attribute is empty, indicating that the decision tree is completed [27].

The establishment of the decision tree is completely dependent on the training samples. If the decision tree grows at will without any restrictions, the decision tree will fit all training samples and make the training accuracy rate reach 100 %. The result of a perfect fit is that the resulting decision tree can only recognize training samples. Overfitting causes the resulting decision tree to be meaningless. Therefore, it is important to prune the complex decision tree generated by the training set and remove some nodes to solve the overfitting problem. This process is called pruning. Pruning methods are divided into pre-pruning and post-pruning [28].

Pre-pruning refers to the estimation of the division of each node in the process of downward bifurcation of the decision tree. If the division of the current node cannot make the decision tree fit other data well after generation, and the generalization ability is improved, the division is stopped. Besides, the current node is marked as a leaf node [29]. Post-pruning is to generate a complete tree after training on the training set, indicating that the classification rules have been established. Then, the non-leaf nodes can be examined from bottom to top or top to bottom. If the subtree corresponding to the node is replaced by a leaf node to improve the generalization ability, pruning is performed to replace the subtree with a leaf node, otherwise, no pruning is performed [30].

Typically, post-pruning techniques preserve more branches of a decision tree than pre-pruning. Post-pruning starts from the bottom up from the root of the tree, so it has a low probability of underfitting. The generalization ability is often better than pre-pruning. Common post-pruning methods include cost complexity pruning, reduced error pruning, pessimistic error pruning (PEP), and minimum error pruning [31].

The C4.5 algorithm adopts the PEP pruning method. The PEP pruning method determines whether to prune subtrees according to the error rate before and after pruning, so a separate pruning dataset is not required.

#### 2.3.2 Improved C4.5 decision tree

When the C4.5 algorithm builds a decision tree, its classification attribute is determined by the information gain rate in information theory. There are many logarithmic operations in the mathematical expression of the information gain rate. During the execution of the algorithm, the library function will be frequently called to implement the logarithmic operation, which will increase the computation time. For this problem, an improved method for calculating the information gain rate is proposed. The information gain rate of the C4.5 algorithms is simplified by the mathematical Taylor equation and McLaughlin equation to reduce the running time of the algorithm.

The derivative of ln(*x*) when *x* = 0 is meaningless, and the commonly used probability value range under the information gain rate calculation formula is between [0, 1], so the McLaughlin equation of ln(*x*) is used to improve the calculation equation of information gain rate in traditional C4.5, as shown in Eq. (6).

(6) $$lnx\approx \left(x-1\right)-\frac{1}{2}{\left(x-1\right)}^{2}+\frac{1}{3}{\left(x-1\right)}^{3}+\ldots +{\left(-1\right)}^{n-1}\frac{1}{n}{\left(x-1\right)}^{n}$$

When x∈(0,1), there are in Eq. (7):

(7) $$lnx\approx \left(x-1\right)-\frac{1}{2}{\left(x-1\right)}^{2}+\frac{1}{3}{\left(x-1\right)}^{3}$$

First, the base change equation is used to convert the log operation into an ln operation. Then, the logarithmic operation is eliminated, and the purpose of simplifying the calculation formula is achieved through the Taylor series expansion and the simplification of taking approximate values. Besides, the efficiency of building a decision tree is improved. The whole derivation process is as follows.

The conversion equation of information entropy is as follows in Eq. (8).

(8) $$E\left(S\right)=-\sum_{j=1}^{m}\frac{{|S}_{j}|}{\left|S\right|}\log_{2}\frac{\left|S_{j}\right|}{\left|S\right|}=-\frac{1}{\ln\;2\times S}\sum_{j=1}^{m}\left[\left(\frac{S_{j}\left(S_{j}-S\right)\left(11S^{2}+2S_{j}-7S_{j}S\right)}{6S^{2}}\right)\right]$$

Here are the conversion equations of conditional information entropy and split information entropy, they are in Eq. (9) and Eq. (10).

(9) $$E_{A}\left(S\right)=-\frac{1}{\ln\;2\times S}\sum_{j=1}^{m}\sum_{i=1}^{N}\frac{S_{ij}\left(S_{ij}-S_{j}\right)\left(11{S_{j}}^{2}+2S_{ij}-7S_{ij}S_{j}\right)}{6S^{2}}$$

(10) $${SplitInfo}_{A}\left(S\right)=-\frac{1}{\ln\;2\times S}\sum_{j=1}^{m}\left[\left(\frac{S_{j}\left(S_{j}-S\right)\left(11S^{2}+2S_{j}-7S_{j}S\right)}{6S^{2}}\right)\right]$$

Analysis of the improved calculation equation shows that the category information entropy is the same every time the information gain rate value of the condition attribute is calculated. Here, $\frac{1}{\ln\;2\times S}$ is omitted in each part when the equation is simplified. This paper adopts an improved equation when the category conditional entropy is calculated to ensure the classification accuracy of the algorithm. It tries to ensure that the order of the information gain rate of each condition attribute is not changed, and the classification accuracy is not affected. The traditional C4.5 algorithm needs to call functions to perform many logarithmic operations. After improvement, there is no logarithmic operation in the calculation equation of the information gain rate, and only four simple mixed operations are left. Therefore, the call to the library function is reduced, and the operation speed of the algorithm will be greatly improved, which will be verified in the following.

#### 2.3.3 Experimental design

This paper verifies the feasibility of the algorithm in the public database of the University of California, Irvine, and it is applied to the research topic. Seven datasets are selected as experimental data. These datasets are widely used in previous decision support systems. In addition, these seven datasets cover experimental data in different fields, including Letter, Gamma, Haberman, Heart, Liver, Waveform and Soybean. The number and records of categories in each dataset are balanced, and each dataset contains different category information.

In order to make full use of the data when training the model and verify the generalization performance of the model in the testing stage, 80 % of the data is selected as the training sample and 20 % of the data is selected as the test sample. The selection of this proportion is a balance, aiming at avoiding the over-fitting of the model to the training data and ensuring that there are enough independent data to evaluate the model performance. In addition, compared with the ratio of 90 %:10 %, 80 %:20 % pays more attention to the performance evaluation on a relatively large test set, which can better reflect the performance of the model on unseen data. In addition, because dividing the dataset into 80 % training set and 20 % test set has provided enough data for training and can better evaluate the generalization performance of the model on new data, k-fold cross-validation method is not selected to verify the selection results of dataset samples in the experiment, as shown in Table 1.

**Table 1:** Datasets required for the experiment.

Dataset name	Letter	Gamma	Haberman	Heart	Liver	Waveform	Soybean
Number of attributions	16	11	3	75	10	40	35
Number of instances	20000	19020	306	303	583	5000	307

Table 1 shows that the attributes of these seven datasets are diverse, some contain 16 attributes (such as Letter dataset) and some only contain 3 attributes (such as Haberman dataset), and the categories involved are also different.

In the comparison process, the traditional C4.5 algorithms and the improved C4.5 algorithms are used respectively. Also, the classification accuracy and execution time are recorded.

### 2.4 Sales prediction model based on NN

Among many neural network algorithms, because BPNN is a multi-layer perceptron structure based on its BPNN, it can realize effective back propagation of errors in network training. Through this algorithm, the nonlinear relationship of sales data can be better approached and the accuracy of sales forecast can be improved. Moreover, BPNN shows good performance on the sales model on the experimental dataset, and its algorithm flow is clear, and the specific implementation steps are clearly presented through the chart. Therefore, this paper chooses BPNN to build a sales forecasting model to make full use of its powerful nonlinear approximation ability to build a more accurate and reliable sales forecasting model.

#### 2.4.1 BPNN

BPNN is a kind of multi-layer perceptron network. The biggest feature of its algorithm is that it can realize the BP of errors in the process of network training. When the training output value is not equal to the actual value, the error is obtained by calculation, and the error is passed from back to front layer by layer [32].

**Fig. 4:**
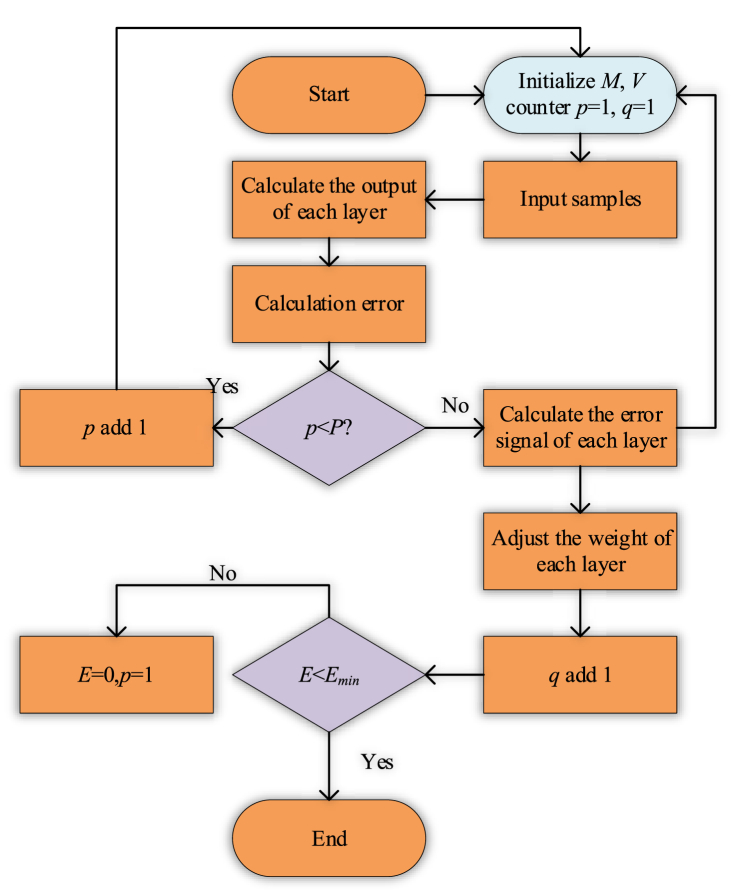
Flow chart of BP neural algorithm.

According to the above implementation steps, the BPNN algorithm is designed. Fig. 4 shows the specific implementation process of each step.

Fig. 4 shows that the steps of BP neural algorithm are as follows. Step 1, initialize *M* and *V* so that *p* = 1 and *q* = 1; Step 2, input samples; Step 3, calculate the output value of each layer; Step 4, calculate the error; Step 5, judge whether *p* is less than the threshold value *P*, if the judgment is successful, calculate the error of each layer, otherwise return to the first step; Step 6, update and adjust the weight of each layer; Finally, output the sample.

#### 2.4.2 Overall model building

**Fig. 5:**
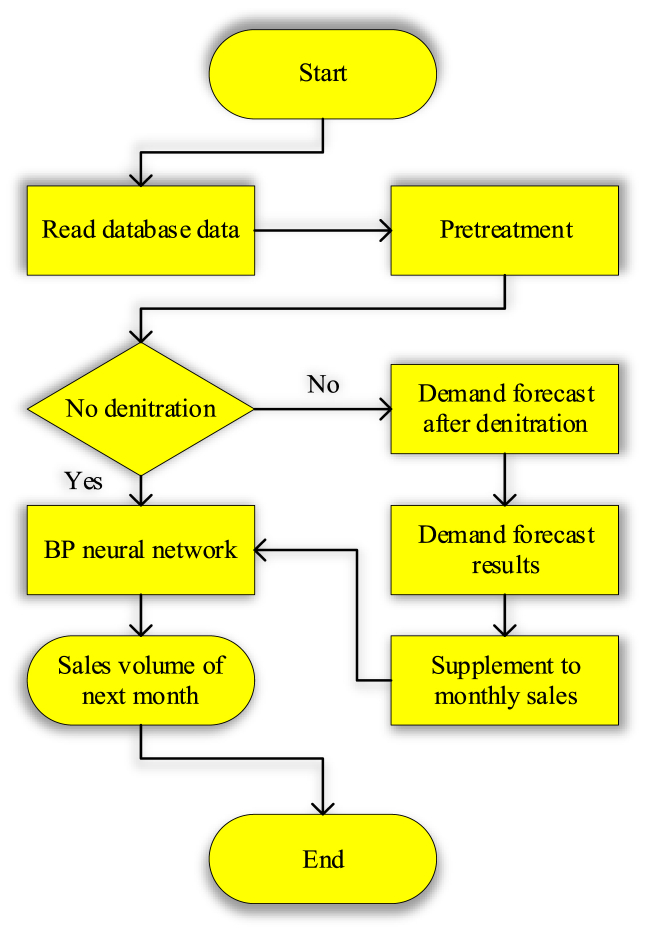
Prediction model flowchart.

This paper uses the strong nonlinear approximation ability of the BPNN to predict it to improve the accuracy of the sales forecast. Fig. 5 shows its prediction model.

Fig. 5 shows that the process of the prediction model constructed in this paper is as follows: Step 1, read the database data; Step 2, preprocess the data; Step 3, judge whether the goods are out of stock, if so, use BPNN to predict the sales volume of next month, otherwise, predict the demand after out of stock; Step 4, supplement the monthly sales.

#### 2.4.3 Experimental design

The actual sales data has seasonal fluctuations with an annual cycle, so it is considered to use a NN with a good nonlinear fit to predict it. Monthly bottled water sales data from 2018 to 2021 is used as a training sample. This part of the data samples is obtained after the data correction scheme proposed here is added to the monitoring data. Furthermore, sales data in 2022 is selected as a test sample.

The parameter settings are as follows. Trainlm is the network training function. A three-layer network is selected, with 48 input layer nodes and one output layer node. The number of training times is set to 1000, the target error is 0.001, and the learning rate η is 0.05.

### 2.5 Design of DSS for the vending machine

#### 2.5.1 Decision support function design

The lightweight vending machine special DSS designed here has three functions: data collection and storage, interactive data analysis, and HCI interface [33]. 1) The data collection is realized by the remote monitoring platform, and the storage is mainly to classify and store the data from the low computer into the corresponding database Table 2) Interactive data analysis refers to the visualization of the data analysis process. The software user can select the appropriate model and adjust the corresponding model parameters in the analysis process according to the original data and the analysis results and analyze the corresponding data. 3) HCI interface means that the results of data analysis are presented graphically, which is convenient for users to obtain the result information intuitively, thereby assisting decision-making [34].

**Table 2:** Product information sheet.

Product attribute name	Number of decimal places reserved	An empty set	Character length	Category
Goods_ID	0	No	10	char
Goods_Name	0	No	20	char
Costs	2	No	2	decimal
Trans_ID	0	No	10	char

**Fig. 6:**
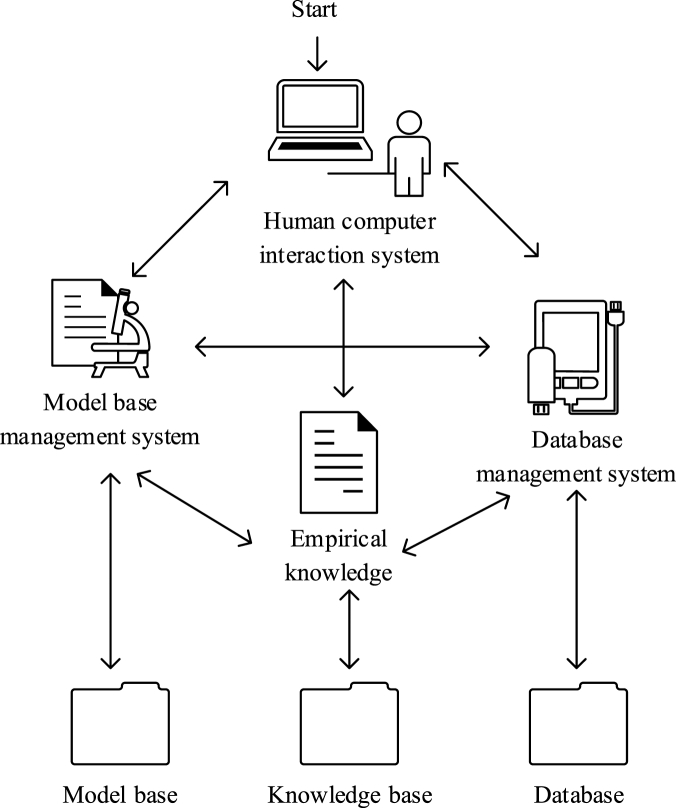
DSS structure diagram.

The structure diagram of the DSS is presented in Fig. 6.

#### 2.5.2 Design of information management module

##### 2.5.2.1 Data management module design

Using the database for data management can isolate the data from the application program, with high development efficiency and easy post-maintenance. The analysis of the existing software and hardware environment reduces the repetitive phenomenon in the development to realize the shared data and greatly improve the utilization of the data in the development. The overall system framework is designed mainly consists of two parts. The first is to receive the data transmitted from the low computer and process the data accordingly. The filtering operation is mainly performed first. Then, the data is analyzed and processed, and the packaging is performed. Therefore, the concise and reliable organization and management of data are realized. The second is to facilitate the management of related transactions in the database. This module needs to check the addition and deletion of data to ensure the relative logical independence and physical independence of the database, providing a strong basis for subsequent decision-making and judgment.

##### 2.5.2.2 Database table design

The data of this paper comes from the experiments and a reliable vending machine management system. In this paper, through the development and use of vending machine management system, relevant data tables are obtained, including replenishment personnel management table, vending machine surplus goods table, cost and inventory table and fault information table. The data table provides detailed information about vending machines for this paper, which enables this paper to conduct further research and analysis. First, the commodity information table is one of the data tables, which mainly records the basic information of the commodities on the vending machine shelves, as shown in Table 2. The table records the basic information such as the name, type, price and inventory of each commodity, which provides an important data source for this paper to study the sales situation of vending machines. This paper ensures the accuracy and reliability of the data, and makes appropriate references and explanations.

The sales information table records sales data. The data packets sent by the low computer are analyzed. According to the product category, the sales are counted every 15 min. The purpose is to reduce the query statistics processing time during data analysis. It includes the following fields: item name, sales volume, and time. The specific design is revealed in Table 3.

**Table 3:** Sales information sheet.

Attribute name	Number of decimal places reserved	An empty set	Character length	Category
Goods_Name	0	No	20	varchar
Goods_Sales	0	No	11	int
Time	0	No	0	datetime

The transaction information table records the basic information of each transaction, and the transaction number is unique. The vending machine number and the commodity number are respectively connected to the vending machine information table and the commodity information table as foreign keys, corresponding to the specific vending machine and commodity. The shelf number can reflect the sale of goods from the specific location of the vending machine. It can not only read the sales price of the goods correspondingly but also provide a good data basis for future research on the theory of vending machine space allocation. The specific design is shown in Table 4.

**Table 4:** Transaction history table.

Attribute name	Number of decimal places reserved	An empty set	Category	Character length
Trans_ID	0	No	char	10
VM_ID	0	No	char	5
Shelf_ID	0	No	char	3
Goods_ID	0	No	char	10
Sell_Time	0	No	datetime	0
Price	2	No	decimal	2

#### 2.5.3 Product sales prediction model design

Requirement analysis is carried out from function and performance realization. A large amount of statistical knowledge is used for predictive modeling under the principles of achieving accuracy, stability, and safety. As a mainstream development language, Java is good at system development. It is also open-source and free, with very powerful functions. This paper is expected to use Java as the server-side development language, but mathematical modeling and computing power are not its strengths. The R language is a powerful tool for statistical computing, and it is also open-source and free. Therefore, the combination of R and Java language is proposed here, which can make up for the shortcomings of Java development and realize modeling and calculation. Java is responsible for the construction of the system, and R is used as the computing engine. R-Java method realizes the call between the two, analyzes the data, and operates in the application program.

Based on the above contents, Java and R language platforms are finally selected to realize the decision support system. Among them, Java, as the main server, is used as the development language to build the system, while R language, as a powerful tool for statistical calculation, is used as the calculation engine. This combination makes use of the advantages of Java in system development, and meanwhile, by integrating the statistical calculation ability of R language, it makes up for the shortcomings of Java in mathematical modeling and calculation, and realizes comprehensive data analysis and predictive modeling functions. Through this software, DSS has realized the functions of data collection and storage, interactive data analysis and HCI interface, providing users with intuitive decision support.

In addition, to improve the comprehensive performance of this system, this paper designs to use reinforcement learning technology to optimize the model comprehensively to improve the product classification effect of the model and improve the efficiency of the machine. The algorithm of the optimization model given in this paper is described as follows.

Step 1 Define states and actions, store action value function by initializing Q-table, and define parameters such as rewards function, learning rate (alpha), discount factor (gamma), and exploration rate (epsilon).

Step 2 Use Q-learning algorithm to update the Q value iteratively, select the action through epsilon-greedy strategy, execute the action, observe the reward and the next state, and then update the Q value;

Step 3 Repeat the above process in multiple episodes until the specified number of iterations is reached. Finally, output the optimal action in each state.

The BPNN and reinforcement learning used in this paper have some similarities in different aspects, but also have some complementary and different advantages and limitations. First, BPNN performs well in supervised learning tasks, and can learn and express complex nonlinear relationships. BPNN optimizes gradient descent through back propagation algorithm, which can adapt to various problems and fit various data. However, BPNN is easy to over-fit, and it takes a long training time for large-scale and complex datasets, and parameter selection and adjustment are relatively complicated. In contrast, reinforcement learning is a learning method in unsupervised learning and unknown environment. It gets reward signals by interacting with the environment, thus gradually optimizing strategies and decisions. Reinforcement learning can adapt to dynamic environment and continuous state space, and has high flexibility and adaptability. However, reinforcement learning requires a long exploration time and computing resources, and it needs to balance the trade-off between exploration and utilization in the training process. On the whole, BPNN and reinforcement learning can complement each other. BPNN is suitable for the problems of supervised learning and known labeled data, while reinforcement learning is suitable for the problems of unsupervised learning and unlabeled data. In practice, BPNN can be used to predict and classify, and then it can be combined with reinforcement learning to optimize decision-making and strategy selection. For example, in product sales forecast, BPNN can be used to forecast sales, and then reinforcement learning method can be used to optimize pricing strategy or inventory management to achieve better sales results. When using BPNN and reinforcement learning, it is necessary to consider the quality and integrity of data, reasonably select the framework, parameters and optimization algorithm of the model, and make repeated adjustments and optimizations in practice. Integrating these two methods can make full use of their advantages in dealing with different problems and situations, thus improving the prediction accuracy and decision-making effect.

## 3 Empirical analysis and data inspection

### 3.1 Product classification model effect based on decision tree algorithm

In this paper, the decision tree algorithm of C4.5 and its optimized algorithm are tested on seven different experimental datasets, and the distribution of the datasets is balanced. In order to evaluate the performance of the model, the experimental indicators are mainly the accuracy, recall, and F1 value of the algorithm. Accuracy is obtained by dividing the number of correctly classified samples by the total number of samples; Recall represents the proportion of samples that are actually positive cases that are correctly predicted by the model; F1 score considers both accuracy and recall [35]. In addition, in order to further measure the computing performance of the algorithm, the timer function in Java software is used to calculate the running time of the algorithm.

**Fig. 7:**
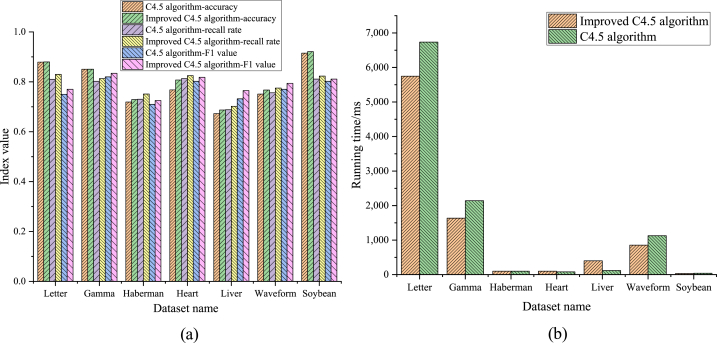
Comparison of the results of the two algorithms on each dataset attribute (a) Comparison of the accuracy of the two algorithms on the attributes of each dataset; (b) Comparison of the running time of the two algorithms on the attributes of each dataset.

The C4.5 decision tree algorithm and the improved C4.5 algorithms are applied to various experimental datasets, and the results obtained are demonstrated in Fig. 7.

In the seven datasets in Fig. 7(a), the highest accuracy of the C4.5 algorithm is about 87 %, and the lowest is about 68 %. The accuracy of the improved C4.5 algorithm is about 87 % at the highest and 67 % at the lowest, and the accuracy of each dataset is lower than about 1 % before the improvement. The two algorithms have the highest accuracy on the Soybean dataset and the lowest accuracy on the Liver dataset. In Fig. 7(b), the running time of the C4.5 algorithm is about 6700 ms at the highest and close to 1 ms at the lowest. The running time of the improved C4.5 algorithm is up to about 5500 ms, and the lowest is close to 1 ms. The running time on all seven datasets is better than the unimproved algorithm. To sum up, there are differences in the accuracy and running time of C4.5 and the improved C4.5 on various dataset attributes. Although there are differences in the accuracy rates of the two algorithms in different datasets, the differences are small. In the running time, the improved C4.5 algorithm is better than the C4.5 algorithm.

### 3.2 Effect of the sales prediction model based on NN

In order to verify the performance of the sales forecasting model based on BPNN, its performance is compared with SVM model and decision tree model, and the comparison results are shown in Table 5.

**Table 5:** Comparison of sales forecast results of different ML models.

Time/model name	Actual value	BPNN	Decision tree	SVM
2018.01	1688.7294	1679	1701	1704
2018.06	1755.47138	1760	1765	1777
2018.12	2205.05494	2186	2011	2022
2019.01	2539.80595	2534	2566	2556
2019.06	2807.52702	2807	2798	2758
2019.12	3276.29364	3252	3245	3145
2020.01	3467.43753	3433	3327	3227
2020.06	3495.94631	3452	3356	3256
2020.12	3237.32943	3189	3495	3463
2021.01	2720.33936	2658	2900	2854
2021.06	2289.58444	2270	2324	2232
2021.12	1935.3624	1973	2001	2028

Table 5 shows that different ML models have certain differences in the prediction of sales volume. Compared with SVM and decision tree model, the sales forecast result of BP model is the closest to the actual value, which shows that BP model has lower forecast error and better forecast accuracy. This reveals that the BPNN prediction model reported here has good accuracy for the sales forecast of vending machines.

### 3.3 Performance comparison of reinforcement learning optimization model

**Fig. 8:**
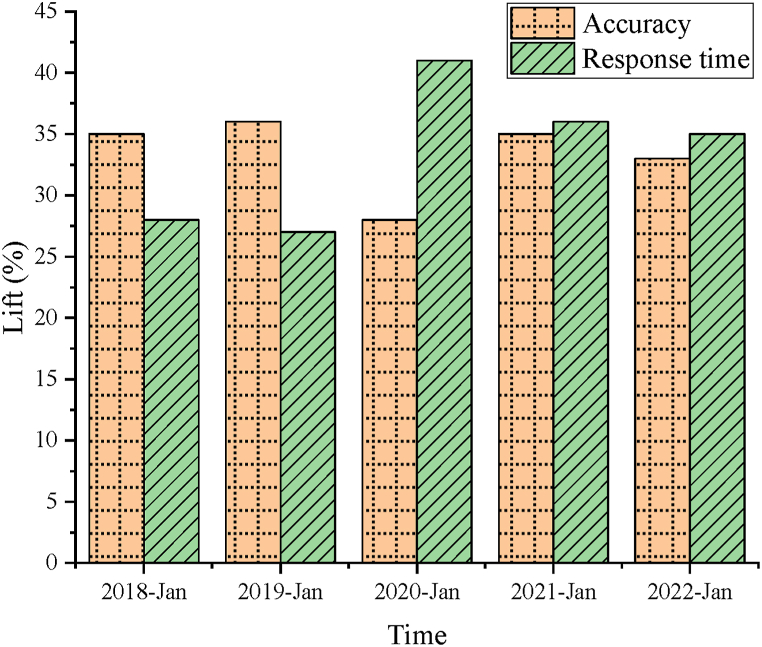
Performance comparison of optimization model.

In order to improve the comprehensive performance of the system designed here, this paper uses reinforcement learning technology to optimize the system, and evaluates and compares the performance of the optimized model with the basic model, which provides important support for optimizing the system in this paper. Fig. 8 shows the performance comparison results of the optimized model.

In Fig. 8, this paper compares the performance of the basic system supported by the designed BP basic model with the system model optimized by reinforcement learning. By comparison, it is found that the accuracy of the system model after reinforcement learning is improved by more than 27 % and the reaction time is improved by more than 29 %, which provides an important support for the performance improvement of the system. In addition, this paper is compared with the current advanced studies, Fei et al. (2021)'s research [36], Maqsood et al. (2022)'s research [37], and Chen et al. (2023)'s research [38]. This paper not only designs a more perfect basic NN model, but also uses a more advanced technical model to upgrade and optimize the basic system model, which means that this paper not only achieves a technological breakthrough, but also provides an important support for the comprehensive upgrade of the system.

### 3.4 Discussion

The above experimental results highlight the potential role of ML in system optimization, especially in the fields of product classification and sales forecast. The application of C4.5 decision tree algorithm reveals the effectiveness of the improved algorithm in improving accuracy and reducing running time, and provides new insights for the application of rule-based ML method in HCI. The excellent prediction performance of BPNN emphasizes the potential value of deep learning in processing time series data and sales trend analysis, which provides strong support for real-time decision-making of vending machines and other systems. The introduction of reinforcement learning has further promoted the improvement of system performance and laid a solid foundation for the comprehensive upgrade of intelligent systems. These results are not only of great significance to the development of technology, but also provide inspiration for the innovation and application in the field of HCI in the future, emphasizing the synergy of ML technology at different levels to achieve a more intelligent and efficient system.

## 4 Conclusion

ML is regarded as technical support. Taking vending machines as an example, the product classification and sales forecast model of vending machines is established by using C4.5 decision tree algorithm and BPNN, and the model is comprehensively optimized by using reinforcement learning technology. On this basis, the theoretical construction of vending machine DSS is completed. The results show that the accuracy of the improved algorithm is almost as high as 90 %, and the minimum running time is close to C4.5. Although the accuracy of the two algorithms is different on different datasets, the difference is very small. The running time of the improved C4.5 algorithm on different datasets is shorter than that of the unmodified algorithm. The prediction curve of BPNN coincides with the curve of actual data, which shows that the sales forecast of vending machines has good accuracy. Meanwhile, through the reinforcement learning technology optimization, the performance of the model is improved by more than 27 %, which shows that this paper has achieved a great technological breakthrough. However, there are some limitations. The DSS of vending machines is only a theoretical structure, and it is not realized by algorithms. In the future, when the amount of data is large, a single machine may not be able to bear the data pressure. Therefore, it is necessary to adopt a distributed architecture to manage data and file structures in a distributed manner to adapt to the rapidly growing amount of information. This will be the content of further research.

## Funding

The work was supported in part by Scientific Research Project of Hunan Provincial Department of Education (No.23A0659). The work was also supported by Research on the gain effect of school-enterprise collaborative education in the mixed teaching mode ——Using “Computers” Taking the course as an example (Grant No.ND233163). The work was also supported by Research and Practice on the Online/Offline Mixed Golden Course Teaching - "Linux Operating System" Based on Superstar Platform (No.HNJG-2021-0217).

## CRediT authorship contribution statement

**Ping Li:** Writing – original draft, Visualization, Data curation. **Fang Xiong:** Methodology, Investigation, Formal analysis. **Xibei Huang:** Validation, Software, Resources. **Xiaojun Wen:** Writing – review & editing, Methodology, Formal analysis.

## Declaration of competing interest

The authors declare the following financial interests/personal relationships which may be considered as potential competing interests:Ping Li reports was provided by Scientific Research Project of Hunan Provincial Department of Education. If there are other authors, they declare that they have no known competing financial interests or personal relationships that could have appeared to influence the work reported in this paper.
